# Posttraumatic stress disorder symptoms in adults with congenital heart disease (ACHD) during the COVID-19 pandemic in Norway

**DOI:** 10.1016/j.ijcchd.2025.100585

**Published:** 2025-04-10

**Authors:** T.K. Grimholt, O. Gjesdal, T. Bonsaksen, T. Heir, O. Ekeberg, I. Schou Bredal, L. Skogstad, I.M. Tøllefsen

**Affiliations:** a Department of Acute Medicine, Oslo University Hospital, Oslo, Norway; b Institute of Nursing, Faculty of Health Studies, VID Specialized University, Oslo, Norway; c Department of Cardiology, Oslo University Hospital, Oslo, Norway; d Department of Health and Nursing, Faculty of Social and Health Sciences, Inland Norway University of Applied Sciences, Elverum, Norway; e Department of Health, Faculty of Health Studies, VID Specialized University, Stavanger, Norway; f Norwegian Center for Violence and Traumatic Stress Studies, Oslo, Norway; g Institute of Clinical Medicine, University of Oslo, Oslo, Norway; h Psychosomatic and CL Psychiatry, Division of Mental Health and Addiction, Oslo University Hospital, Oslo, Norway; i Department of Public Health Science, Faculty of Medicine, Institute of Health and Society, Faculty of Health Sciences University of Oslo, Oslo, Norway; j Department of Health and Care Sciences, UiT The Arctic University of Norway, Tromsø, Norway

**Keywords:** Congenital heart disease, COVID-19, Survey, Symptom defined posttraumatic stress disorder

## 1 Introduction

Compared to the general population, people with Adult Congenital heart defect (ACHD) were defined as a potentially vulnerable group if infected by Severe Acute Respiratory Syndrome Coronavirus 2 (SARS-CoV-2) during the initial phase of the pandemic. The group has increased risk of complications, for arrhythmias, heart failure, pulmonary hypertension, and premature death [1], but the clinical response to SARS-CoV-2 infection was unknown. The estimated prevalence of ACHD is about 3000 per million with approximately 2.3 million in Europe [2].

The outbreak of the Corona virus was declared as a pandemic by the World Health Organization in January 2020 [3]. Two months later, the Norwegian government initiated a lockdown with several intrusive interventions for the population to minimize the contagion of the virus, morbidity, and mortality and thus hinder a breakdown of the public health care services. Schools were closed, social contact and travelling restricted. COVID-19 led to many hospital admissions and placed a significant burden on the health care services especially the Intensive Care Units worldwide. Six months after the outbreak, more than 3.3 million cases with >190 000 deaths have been counted by the European Centre for Disease Prevention and Control [4].

In addition to excess mortality rates and hospital admissions, several studies demonstrated mental health problems and concerns in the general population related to psychosocial health like insomnia, general worries about the future, anxiety, depression, and post-traumatic stress [[5], [6], [7], [8]].

Cardiologists posed theories that respiratory diseases like hypoxia or embolism caused by the virus in people with complex heart defects could have adverse effects and potentially be lethal [9]. Further, cardiologists experienced that patients were afraid to be infected and therefore postponed scheduled appointments in the specialist health services [10]. It was also anticipated that the mental health of many patients would be challenged by stressors like social isolation, fear of contracting COVID-19, frustration with the actions of others, concern about seeking cardiology care (both routine and urgent), adaptation to telemedicine, thoughts of mortality, and implications for finances, education, and employment [9]. Previous research has demonstrated that more mental health issues, included symptoms of PTSD occur in the ACHD population compared to the general population [11].

Being chronically ill and continuously adapt to a life marked by outpatient follow-up, repeated hospitalizations, invasive medical procedures, and persistent fear and uncertainty are known factors [12] This lifelong exposure to medical stressors may increase vulnerability to symptoms of post-traumatic stress disorder. Given these vulnerabilities, it was plausible that external stressors such as the COVID-19 pandemic, and particularly the fear of infection and premature death raised the question of whether such experiences could contribute to symptoms of post-traumatic stress disorder like, including intrusive memories avoidance behaviors, emotional numbing, and heightened arousal.

However, there are to our knowledge little studies of mental health and concerns in these groups concerning the fear of COVID-19 infection and potential lethality.

### 1.1 Aims

The aims were to study symptom defined post-traumatic stress disorder, self-assessed anxiety and depression, and concerns about COVID-19 in Norwegian adults with congenital heart disease compared to the general population during the COVID-19 pandemic. Further, the aim was to compare simple vs. complex CHD on these measures.

## 2 Materials and methods

An electronic survey was disseminated through the Union of Grown-Ups with Congenital Heart disease in Norway (GUCH). The link to the survey was shared in emails and on Facebook during the period May–October 2020. Age criteria for inclusion was ≥18 years.

An electronic survey was conducted in the general Norwegian population (Corona-Pop) in April and May 2020. A snowball sampling strategy was utilized. The survey was disseminated by Oslo University Hospital, University of Oslo, and Oslo Metropolitan University. The link to the survey was published on social media such as Facebook, Twitter, and Instagram. The study was also featured in national and local newspapers with online links. Norwegian citizens aged 18 years or older were invited to participate in the survey. There were no other exclusion criteria.

In both surveys, The University of Oslo “Nettskjema”, which is a tool for designing and conducting online surveys, was used and the data were stored using the Services for sensitive data.

### 2.1 Ethics

The studies leading to this paper were approved by the Regional Committee for Medical and Health Research Ethics in Norway. (ID:144147/130447) and the Personal Protection Agency at Oslo University Hospital. This included waived need for informed consent since the survey was anonymous.

### 2.2 Patient and public involvement

The Union of Grown-ups with Congenital Heart Disease in Norway contributed to disseminate the survey and will publish the results to their members.

### 2.3 Measures

The study instruments and questions were similar to those previously employed in studies of the Norwegian population in 2015 (NorPop-study) [13,14] and during the first wave of the Corona outbreak in 2020 (Corona-pop) [8].

#### 2.3.1 Sociodemographic variables

Data were collected for age groups (18–29 years, 30–39 years, 40–49 years, 50–59 years, 60–69 years, and 70 years or older), gender (male/female), highest completed educational level (high school or lower versus higher education), current employment status (working/paid leave/in education versus not), marital status (living with spouse or partner versus not), and size of place of residence (<200 inhabitants, 200–19.999 inhabitants, 20.000–99.999 inhabitants, 100.000 inhabitants or more).

#### 2.3.2 Heart specific variables

Congenital heart defect (CHD) diagnoses were based on The International Classification of Diseases (ICD-10) and were listed with a yes/no response included an open box with free text in the survey. If more than one CHD was reported, the most severe diagnosis was used. CHDs was defined as a structural defect in the heart or the great vessels that was present at birth. According to similar studies, the sample was divided into simple and complex defects (Table 1) [15]. Questions about number of heart surgeries (continuous scale), pacemaker, valve replacement (yes/no) and heart disease last month (yes/no) were added.

**Table 1:** Diagnosis hierarchy congenital heart defect^a^.

Complex
1. Univentricular hearts (UVH)
2. Truncus arteriosus communis (TAC)
3. Interrupted or hypoplastic aortic arch (I/HAA)
4. Transposition of the great arteries (TGA)
5. Atrioventricular septal defect (AVSD)
6. Totally anomalous pulmonary venous drainage (TAPVD)
7. Pulmonary atresia (PA)
8. Tetralogy of Fallot (TOF)
*Simple*
9. Ventricular septal defect (VSD)
10. Coarctation of the aorta (CoA)
11. Aortic stenosis (AS)
12. Pulmonary stenosis (PS)
13. Mitral valve defect (MV)
14. Partially anomalous pulmonary venous drainage (PAPVD)
15. Atrial septal defect (ASD)
16. Patent ductus arteriosus (PDA)
17. Other (MISC)

a The Hierarchy Congenital Heart Defect table is based on Erikssen, G. et al. [15].

#### 2.3.3 The PTSD checklist for DSM-5 (PCL-5)

Questions about current posttraumatic stress symptoms associated with the COVID-19 outbreak were used. To achieve a possible PTSD diagnosis, a respondent had to fulfil the DSM-5 symptom criteria for PTSD, except from the A criterion (having experienced accidental or violent death, threat of life, serious injury, or sexual violence) [16]. We used survey data on the PTSD Checklist (PCL-5) to measure symptoms. The 20-item PCL-5 is a self-administered questionnaire assessing the full domain of the DSM-5 PTSD diagnosis [17]. The instrument has four subscales, corresponding to each of the symptom clusters in the DSM-5. The symptoms endorsed were specifically linked to the COVID-19. Each item was scored on a 5-point scale (0 not at all, 1 a little, 2 moderately, 3 quite a bit, 4 extremely) to rate the extent to which the relevant symptom had bothered the person during the past month.

DSM-5 diagnostic guidelines [16] were applied to the PCL-5 to categorize participants as fulfilling PTSD symptom criteria or not. Participants indicating a score of 2 or above on at least one of five re-experiencing symptoms, one of two avoidance symptoms, two of seven symptoms of negative alterations in cognition and mood, and two of six arousal symptoms were classified as fulfilling the PTSD symptom criteria [17,18]. The Norwegian version of the PCL-5 was developed through an alternating procedure of translations and back-translations [19]. The original authors approved the final English back-translation. PCL-5 has good or excellent internal consistency, reliability, and validity [17,18,20].

#### 2.3.4 Anxiety and depression

The questionnaire included the question: “Below is a list of health problems: Do you have, or have you had, any of these?” Among the listed problems were anxiety and depression. The answer options were: “no”, “yes”, and “last month” (i.e., during the COVID-19 lockdown).

#### 2.3.5 COVID-19 specific questions

The problems related to COVID-19 were assessed with a dichotomous scale yes/no whether they had economic concerns, general worries, had been infected with the coronavirus and self-identified themselves as being at risk of complications.

### 2.4 Statistical analyses

Cases with positive scores on the critical number of items in each symptom cluster were considered symptom-defined PTSD. Those that could not have reached the critical number of positive items regardless of scores on missing items were considered non-symptom-defined PTSD. Categorical variables were analyzed using chi-square test. A 2-sided p-value <0.05 was considered statistically significant. All analyses were performed by IBM SPSS software, version 26.0 (SPSS Inc.Chicago IL).

## 3 Results

### 3.1 Sample characteristics

The total sample included 283 ACHD patients and 4527 adults from the general population. In total there were more females than males. Age and marital status were equally distributed. In the ACHD group, the educational level was lower, and a larger proportion of people received disability benefits. A higher proportion of ACHD patients lived in rural areas (Table 2).

**Table 2:** Sociodemographic characteristics in the ACHD and General Population (GP) samples.

Variables	ACHD (n = 283)	GP (n = 4527)	P-value
	n (%)	n (%)	
Age group (years) (n = 4810)			0.34
18-19	4 (1.4)	72 (1.6)	
20-29	73 (25.8)	1084 (23.9)	
30-39	76 (26.9	1220 (26.9)	
40-49	69 (24.4)	931 (20.6)	
50-59	46 (16.3)	766 (16.9)	
60-69	13 (4.6)	354 (7.8)	
70-79	2 (0.7)	82 (1.8)	
80>	0	18 (0.4)	
Gender (n = 4790, 20 missing gender)			<0.001
Male	86 (30.6)	659 (14.6)	
Female	195 (69.4)	3850 (85.4)	
Marital status (n = 4810)			0.21
Widower	2 (0.7)	48 (1.1)	
Married/engaged	177 (62.5)	2716 (60.0)	
In a relationship	18 (6.4)	318 (7.0)	
Divorced	4 (1.4)	187 (4.1)	
Single	82 (29.0)	1258 (27.8)	
Education (n = 4805, 5 missing)			<0.001
Primary school	16 (5.7)	117 (2.6)	
High school	104 (36.7)	988 (21.8)	
University/college <4 years	83 (29.3)	1376 (45.1)	
University/college >4 years	80 (28.3)	2041 (45.1)	
Employment status (now) (n = 4804, 6 missing)			<0.001
Working	170 (60.3)	3242 (71.7)	
Military service	0	5 (0.1)	
Under education	21 (7.4)	425 (9.4)	
Unemployed	15 (5.3)	349 (7.7)	
Disability	68 (24.1)	297 (6.6)	
Retired	8 (2.8)	204 (4.5)	
Size of place or residence (n = 4800, 10 missing)			<0.001
Village <200 inhabitants	25 (8.8)	187 (4.1)	
Village/city 200–19.999 inhabitants	86 (30.4)	1141 (25.3)	
City 20.000–100.000 inhabitants	86 (30.4)	1091 (24.2)	
City >100.000 inhabitants	86 (30.4)	2098 (46.4)	

In the ACHD group, 145 patients (51 %) had complex CHD and 138 (49 %) had simple CHD. There were no significant differences on sociodemographic characteristics, pacemaker or valve replacement between the complex vs. simple CHD groups. There were significantly more people that had gone through heart surgery yes/no in the complex ACHD group compared with simple ACDH (95.9) vs. 86.2 %, p = 0.004) (Table 3).

**Table 3:** Severity of congenital heart defect (CHD) and related heart disease.

Variables	All (n = 283)	Complex CHD n = 145	Simple CHD n = 138	P-value
	n (%)	n (%)	n (%)	
Heart surgery				0.004
Yes	258 (91.2)	139 (95.9)	119 (86.2)	
No	25 (8.8)	6 (4.1)	19 (13.8)	
Number of heart surgery^a^ n = 276				<0.001
0–1 surgery	119 (43.1)	42 (29.4)	77 (57.9)	
≥ 2 surgeries	157 (56.9)	101 (70.6)	56 (42.1)	
Pacemaker^b^ n = 279				0.42
Yes	59 (21.1)	33 (23.1)	26 (19.1)	
No	220 (78.9)	110 (76.9)	110 (80.9)	
Valve replacement c n = 264				0.57
Yes	61 (23.1)	32 (24.6)	29 (21.6)	
No	203 (76.9)	98 (75.4)	105 (78.4)	
Heart disease last month n = 283				0.036
Yes	82 (29.0)	50 (34.5)	32 (23.2)	
No	201 (71.0)	95 (65.5)	106 (76.8)	

a 7 missing.

b 4 missing.

c 19 missing.

### 3.2 Symptom defined PTSD, anxiety and depression

The prevalence of symptom defined PTSD (29.3 % vs. 18.5 %, p < 0.001), anxiety (26.9 % vs. 17.1 %, (p < 0.001) and depression (20.5 % vs. 12.5 %, p < 0.001) last month was significantly higher in the ACHD group compared to the general Norwegian population (Table 4).

**Table 4:** Comparison between ACHD and General Population (GP) on symptom defined PTSD, anxiety, depression, and COVID-19 related concerns.

Variables	ACHD (N = 283)	GP (N = 4527)	P-value
	n (%)	n (%)	
Symptom defined PTSD	81 (29.3)	832 (18.5)	<0.001
Anxiety last month	76 (26.9)	773 (17.1)	<0.001
Depression last month	58 (20.5)	568 (12.5)	<0.001
Economic concerns (yes)	46 (16.4)	985 (21.8)	0.03
Generally worried because of the pandemic	165 (58.5)	2748 (60.9)	0.44
COVID-19 infected	3 (1.1)	65 (1.4)	0.61
Risk group for complications of COVID-19	191 (69.7)	1061 (23.5)	<0.001

There was no significant difference between complex CHD vs. simple CHD patients on symptom defined PTSD (34 % vs. 24.4 %, p = 0.08), anxiety last month (31 % vs. 22.5 %, p = 0.10) and depression last month (22.8 % vs. 18.1 %, p = 0.33).

### 3.3 COVID-19 specific questions

The ACHD group had significantly lower economic concerns (16.4 % vs. 21.8 %, p = 0.03), but defined themselves in risk group of complications for COVID-19 (69.7 vs.23.5 %, p < 0.001). There was no significant difference in general worries.

## 4 Discussion

This study investigated the present of post-traumatic stress symptoms during the first outbreak of COVID-19. We found significant higher symptom defined PTSD among people with ACHD compared to the general Norwegian population. These results were also found in a systematic review of people with general chronic diseases, of the Covid-19 epidemic where loneliness and lack of social support increased post-traumatic stress, depressive and anxiety symptoms [21].

In contrast to previous research that showed associations with worse clinical state of the patient's congenital heart disease and more post-traumatic stress symptoms [22], there was no statistical difference in symptom defined PTSD between patients with complex-vs simple ACHD. However, the group with simple CHD were closer to the general population. This is in line with results that showed that considering oneself to be in risk group for COVID-19 related complications was associated with risk of PTSD [8]. One possible explanation could be that near 70 % of the ACHD patients regardless of complexity considered themselves at risk for complications at an early stage of the pandemic. At this time there were limited knowledge about specific risk factors associated with a complicated COVID-19 course among clinicians and patients with ACHD.

Initially plausible arguments of factors that posed high risk for ACHD patients were discussed by Diller et al. [9]. However, one early cohort study from 2020 observed a milder COVID-19 clinical course with low cardiovascular complications and zero lethality among CHD patients [23]. More specifically, a study demonstrated that congenital cardiac defects at particularly high risk were cyanotic lesions, including unrepaired cyanotic defects or Eisenmenger syndrome [24]. This was supported by a study that found that the most vulnerable patients were those with worse physiological stage, such as cyanosis and pulmonary hypertension, whereas anatomic complexity did not appear to predict infection severity. The authors concluded that COVID-19 mortality in adults with CHD is commensurate with the general population [25].

Adults with congenital heart disease remain chronically ill and must continuously adapt to a life with outpatient follow-up, repeated hospitalizations, invasive medical procedures, and persistent fear and uncertainty [12], and also in non-pandemic times psychiatric disorders, particularly mood and anxiety disorders are significantly more frequent in ACHD compared to the general population [26]. A history of cardiac surgery also increases were significantly posttraumatic stress disorder significantly [27].

In a large study, higher levels of PTSD were found in the CHD group compared to those without CHD (0.8 % vs 0.5 %, respectively). More than one out of ten were diagnosed with an anxiety or depression disorder [28]. The authors underpin the need for treatment and refer to the National guidelines on the management of PTSD. This cover recognizing, assessing, and treating symptoms aimed at improving quality of life by reducing symptoms like sleep problems and anxiety. These recommendations also focus on improvement of coordinated care and especially trauma focused therapy as the first line of treatment [29].

There seems to be a need to address these problems in ACHD patients in general and even more likely during- and post pandemic. In a cross-sectional study of ACHD patients during the pandemic, it was found that 19.5 % reported problems getting necessary medical treatment or visiting a physician. Anxiety and depressive symptoms were associated with medical undertreatment [30]. In UK, the telephone advice service at a level 1 Centre showed a significant increase in ACHD patients seeking advice when compared with 2019 the year before the pandemic. There were also large peaks following major Government announcements [10].

We agree with the recommendations by Wray, that service delivery and support during the pandemic may have improved patients' experience of care [31].

In recent years telemedicine has emerged as a strategy to increase access to subspecialty care. US researchers found that ACHD patients were satisfied with virtual visits [32]. Similar results were found during the COVID-19 pandemic, where the use of telemedicine increased and showed positive experiences in ACHD patients [33].

To prepare for a new outbreak, policymakers and clinicians should consider permanent strategies like screening and referral for services to ensure sufficient psychosocial help. Such strategies might enhance the health care services to this group also in non-pandemic times. Especially digital health care could be beneficial for people in rural areas with long travel to receive health care, and for those who are afraid of being infected of e.g., influenzae or other potentially harmful virus diseases.

Further research should investigate mental health issues and need for psychosocial help among people with ACHD. Given the exploratory nature of this study and the limited sample size in the ACHD group, we restricted analyses to bivariate comparisons to avoid overfitting and unstable estimates in multivariable models [34,35]. However, there appears to be a need to investigate the independent contributions of various sociodemographic and psychosocial factors to mental health outcomes in this population through adequately powered multivariable analyses. Longitudinal studies could also help clarify causal pathways and inform the development of targeted interventions.

This study has several limitations that should be mentioned. First, PTSD symptoms were assessed using self-report questionnaires rather than clinical diagnostic interviews, which may affect diagnostic accuracy. Second, the cross-sectional design limits our ability to draw conclusions about causality. Third, the general population sample included a higher proportion of females, younger individuals, and those with higher education levels, suggesting potential selection bias. Fourth, the use of an anonymous online survey precluded follow-up or comparison with non-responders. Fifth, the data collection periods differed between groups: ACHD participants were surveyed between May and October 2020, while the general population data were collected in April–May 2020. Given the rapidly changing public health context early in the pandemic, this timing difference may have influenced symptoms of posttraumatic stress. Finally, the ACHD sample was recruited through a national patient organization, which may have introduced additional selection bias. Individuals affiliated with such groups may differ from other adults with CHD in terms of healthcare engagement, experiences, or psychosocial vulnerability. These factors should be taken into consideration when interpreting the generalizability of the findings.

In conclusion people with ACHD reported more posttraumatic stress symptoms than the general population during the outbreak of COVID-19, in particular patients with complex ACHD. These results underpin a need to ensure sufficient measures and a systematic approach for ACHD patients to improve their mental health during critical circumstances such as the COVID-19 pandemic.

## CRediT authorship contribution statement

**T.K. Grimholt:** Writing – original draft, Supervision, Project administration, Methodology, Data curation, Conceptualization. **O. Gjesdal:** Writing – review & editing, Methodology, Investigation, Conceptualization. **T. Bonsaksen:** Writing – review & editing, Methodology, Investigation, Formal analysis, Data curation. **T. Heir:** Writing – review & editing, Supervision, Methodology, Formal analysis, Data curation, Conceptualization. **O. Ekeberg:** Writing – review & editing, Validation, Supervision. **I. Schou Bredal:** Writing – review & editing, Visualization, Supervision, Investigation. **L. Skogstad:** Writing – review & editing, Investigation. **I.M. Tøllefsen:** Writing – original draft, Validation, Formal analysis, Data curation.

## Availability of data and materials

The datasets used and/or analyzed during the current study are available from the corresponding author on reasonable request.

## Ethics approval and consent to participate

No person-identifying information was collected. Those who consented to participate did so by answering the electronical survey. The study was approved by the Regional Ethics Committee of Health South-East and the requirement for informed consent waived because the data was collected anonymously.

## Consent for publication

Not applicable.

## Funding

The authors received no financial support for the research, authorship, and/or publication of this article.

## Declaration of competing interest

The authors declare that they have no known competing financial interests or personal relationships that could have appeared to influence the work reported in this paper.
